# Potential for Controlling Cholera Using a Ring Vaccination Strategy: Re-analysis of Data from a Cluster-Randomized Clinical Trial

**DOI:** 10.1371/journal.pmed.1002120

**Published:** 2016-09-13

**Authors:** Mohammad Ali, Amanda K. Debes, Francisco J. Luquero, Deok Ryun Kim, Je Yeon Park, Laura Digilio, Byomkesh Manna, Suman Kanungo, Shanta Dutta, Dipika Sur, Sujit K. Bhattacharya, David A. Sack

**Affiliations:** 1 Johns Hopkins Bloomberg School of Public Health, Baltimore, Maryland, United States of America; 2 International Vaccine Institute, Seoul, Republic of Korea; 3 National Institute of Cholera and Enteric Diseases, Kolkata, India; Johns Hopkins Bloomberg School of Public Health, UNITED STATES

## Abstract

**Introduction:**

Vaccinating a buffer of individuals around a case (ring vaccination) has the potential to target those who are at highest risk of infection, reducing the number of doses needed to control a disease. We explored the potential vaccine effectiveness (VE) of oral cholera vaccines (OCVs) for such a strategy.

**Methods and Findings:**

This analysis uses existing data from a cluster-randomized clinical trial in which OCV or placebo was given to 71,900 participants in Kolkata, India, from 27 July to 10 September 2006. Cholera surveillance was then conducted on 144,106 individuals living in the study area, including trial participants, for 5 y following vaccination. First, we explored the risk of cholera among contacts of cholera patients, and, second, we measured VE among individuals living within 25 m of cholera cases between 8 and 28 d after onset of the index case. For the first analysis, individuals living around each index case identified during the 5-y period were assembled using a ring to define cohorts of individuals exposed to cholera index cases. An index control without cholera was randomly selected for each index case from the same population, matched by age group, and individuals living around each index control were assembled using a ring to define cohorts not exposed to cholera cases. Cholera attack rates among the exposed and non-exposed cohorts were compared using different distances from the index case/control to define the rings and different time frames to define the period at risk. For the VE analysis, the exposed cohorts were further stratified according to the level of vaccine coverage into high and low coverage strata. Overall VE was assessed by comparing the attack rates between high and low vaccine coverage strata irrespective of individuals’ vaccination status, and indirect VE was assessed by comparing the attack rates among unvaccinated members between high and low vaccine coverage strata.

Cholera risk among the cohort exposed to cholera cases was 5–11 times higher than that among the cohort not exposed to cholera cases. The risk gradually diminished with an increase in distance and time. The overall and indirect VE measured between 8 and 28 d after exposure to a cholera index case during the first 2 y was 91% (95% CI 62%–98%) and 93% (95% CI 44%–99%), respectively. VE persisted for 5 y after vaccination and was similar whether the index case was a young child (<5 y) or was older. Of note, this study was a reanalysis of a cholera vaccine trial that used two doses; thus, a limitation of the study relates to the assumption that a single dose, if administered quickly, will induce a similar level of total and indirect protection over the short term as did two doses.

**Conclusions:**

These findings suggest that high-level protection can be achieved if individuals living close to cholera cases are living in a high coverage ring. Since this was an observational study including participants who had received two doses of vaccine (or placebo) in the clinical trial, further studies are needed to determine whether a ring vaccination strategy, in which vaccine is given quickly to those living close to a case, is feasible and effective.

**Trial registration:**

ClinicalTrials.gov NCT00289224

## Abstract 2

Mohammad Ali, David Sack and colleagues re-analyze data from a cluster randomized controlled trial of oral cholera vaccine to determine the potential effectiveness of a ring vaccination strategy.

## Author Summary

**Why Was This Study Done?:**

- Cholera remains a major health problem in many Asian and African countries and Haiti, and the current oral cholera vaccines approved by the World Health Organization are in limited supply.
- People are at higher risk of contracting cholera when they live near a cholera case, suggesting that a potential strategy to reduce infection rates would be to quickly vaccinate a buffer (ring) population around identified cases.
- There is a need to understand the magnitude of the increased risk for people living near a cholera case and how one might target oral cholera vaccines to those at highest risk to maximize the benefit from vaccination.

**What Did the Researchers Do and Find?:**

- To explore the potential vaccine effectiveness of oral cholera vaccines in a ring vaccination strategy, we analyzed existing data from a previously conducted cluster-randomized clinical trial in which oral cholera vaccine or placebo was given to 71,900 individuals in Kolkata, India, from July through September 2006.
- Out of 144,106 individuals living in the study area, including trial participants, 672 cases of cholera (index cases) were detected between 1 October 2006 and 25 September 2011 and were matched with 672 randomly selected age-matched controls who did not have cholera.
- Individuals living within a certain distance of each index case and index control were used to define clusters of individuals exposed and not exposed to a cholera case. The overall and indirect protective effectiveness of the vaccine were estimated by comparing rates of cholera when a high proportion versus a low proportion of the people in these clusters around index cases had received vaccine.
- Cholera risk was significantly increased for people living within 25 to 50 m of the cases compared to people living within 25 to 50 m of controls without cholera; this risk persisted for a month but decreased over space and time during the month.
- When we compared clusters with high vaccine coverage with those with low coverage, the vaccine had an overall and indirect effectiveness greater than 90% for two years; the effectiveness persisted for five years but waned somewhat after the first two years.

**What Do These Findings Mean?:**

- Cholera risk is increased for neighbors of cholera patients, but this risk is significantly decreased when a high proportion of neighbors have received vaccine.
- If vaccine supply is limited, a strategy of immunizing neighbors around a case (“ring vaccination”) may be beneficial for controlling cholera if such vaccination can be carried out rapidly.
- Future studies are needed to evaluate the feasibility and effectiveness of a ring vaccination strategy in which vaccine is provided to family and neighbors immediately after cases are detected.

## Introduction

Cholera is estimated to infect about 2.8 million individuals and cause 91,000 deaths per year worldwide [1]. The current prequalified oral cholera vaccines (OCVs) provide over 80% (direct) protection in the first 6 mo after vaccination [2–4], with protection of up to 65% for 5 y estimated in an endemic setting [5]. However, this estimate does not include herd protection. When herd protection is taken into account, with coverage of 50%, the disease might be eliminated in endemic areas [6]. Despite this information, there is need to identify potentially effective strategies for reducing transmission and saving lives from cholera. A major limitation for implementation of OCV vaccination stems from the limited number of doses available in the global stockpile [7] as well as limited resources to deliver vaccine to those living in cholera-prone areas.

Cholera cases tend to be spatially aggregated [8–10], with populations near a cholera case at high risk for the disease. This clustering may be related to small common source outbreaks or to fecal-oral transmission within small areas, or to a combination of the two factors. Because of this high risk around cases, a potential strategy to reduce cholera infection rates is to vaccinate a buffer (ring) population around an identified case. This approach could break transmission, and may be a feasible method to achieve high coverage in limited high-risk groups even though the overall population coverage in the endemic area will remain relatively low [6].

Vaccinating a buffer around identified cases was successfully used in the smallpox eradication program and has the potential of reducing the number of doses needed to control a given infectious disease [11]. A ring vaccination strategy was also found to be effective in a recent study conducted in Guinea, in West Africa, against Ebola virus [12]. A similar strategy might be a highly cost-effective method to limit cholera transmission, and may be especially useful in densely populated cities in Africa and in Asia. These cities appear to act as regional hubs of transmission [9], but currently there is not a sufficient vaccine supply to immunize entire urban populations at risk.

In this study, we explored the potential of a ring vaccination strategy to control cholera by leveraging the data from a large clinical trial. We first explored the magnitude of risk around cholera cases at different spatiotemporal scales. Based on this exploration, we identified a suitable scale for a hypothetical ring vaccination strategy in that setting, and estimated the overall and indirect vaccine effectiveness (VE) of OCV using this ring vaccination strategy.

## Methods

This study used existing data from a clinical trial approved by the ethics committee of the National Institute of Cholera and Enteric Diseases, the Health Ministry Screening Committee of India, and the International Vaccine Institute Institutional Review Board. Participants aged >17 y and parents or guardians of participants aged 1–17 y provided written informed consent. Written assent was also obtained from adolescents aged 12–17 y. Thumbprints were obtained and witnessed if the participant, or their guardian, was illiterate. Any patient coming from our study area irrespective of his/her participation in the initial trial gave written informed consent at the time they came to a project clinic/hospital for treatment of diarrhea. We obtained verbal individual household consents as well as community consents to carry out census and demographic surveillance in the study area.

### The Study Data

We analyzed existing data from a cluster-randomized, double-blind, placebo-controlled trial of two doses of an OCV conducted in a densely populated urban slum of Kolkata, India. Vaccine or placebo was given to 71,900 participants during the period 27 July to 10 September 2006 in a study area that encompassed a population of about 110,000 [5]. Cholera surveillance was then conducted on 144,106 individuals living in the study area, including trial participants, between 1 October 2006 and 25 September 2011. In this trial, the clusters were dwellings, which were randomly assigned to receive either vaccine or placebo. Residents who were at least 1 y of age and were not pregnant were eligible to participate in the study. Before the trial, a census was conducted to register the population, map the households residing in the area, assign unique study ID numbers to each individual, and collect information on household socioeconomic, water-use, sanitation, and hygienic characteristics. The study population was subsequently updated by a routine demographic surveillance system.

### Index Cases and Controls

We defined index cases as those who lived in the study area, irrespective of study vaccination arm, who were confirmed to have cholera during the 5-y surveillance period after vaccination in the community. We then selected an index control randomly for each index case from the same population, matching by age group (<5 y, 5 to <15 y, and 15 y and above) at the date of admission of the index case. The index controls did not have cholera from 7 d prior to the onset date of their index case until the end of the surveillance period.

### Exposed and Non-exposed Cohorts

Individuals living around each index cholera case were assembled using a distance buffer (ring) to define a cohort of individuals exposed to the cholera index case. Similarly, individuals living around each index control were assembled using a ring to define a cohort of individuals not exposed to a cholera case. Different spatial scales from 10 to 55 m stepped by 5 m were used to define the cohorts. For each index case we defined ten exposed cohorts for the ten different spatial scales. Similarly, we defined ten non-exposed cohorts for the ten different spatial scales for each index control.

### Spatiotemporal Risk for Cholera

We explored different space and time scales to evaluate the risk of cholera among cohorts exposed and not exposed to index cholera cases. Index cases themselves and index controls themselves were not included as part of the cohorts under follow-up. We measured the cholera attack rates among the exposed and non-exposed cohorts within a specified time frame, *t*_1_ to *t*_2_, and within a specified distance range, *d*_1_ to *d*_2_. We used the following time frames: 0–7 d, 8–14 d, 15–21 d, 22–28 d, 29–35 d, and 36–42 d from the date of onset of index cases. We choose weekly intervals since they can be practical intervals to implement public health interventions.

We calculated the relative risk of being a new cholera case among exposed and non-exposed cohorts using the different specified distances and time frames. We also calculated the risk ratio of cholera between cohorts after adjusting for several covariates found to be significantly associated with the risk for cholera: vaccination status (recipients of two doses of the vaccine versus non-recipients of two doses of the vaccine), age (in years), sex (male versus female), and distance (linear) from household to the nearest water body [5]. Based on evaluation of cholera risk at different spatiotemporal scales, we selected a suitable ring size that may be logistically feasible and cost-effective for a ring vaccination strategy.

### Estimating Vaccine Effectiveness

We built a dynamic population in this analysis and included all individuals residing in the rings around the index cases, including those who participated in the vaccine trial (recipients of vaccine or placebo), those who did not participate in the trial, and those who migrated into or were born in the study area after the vaccination trial was over. We calculated the level of vaccine coverage within the exposed cohorts, taking into account the two-dose recipients and all individuals residing in the rings. To evaluate VE, we compared cholera incidence in cohorts with the highest coverage and cohorts with the lowest coverage. We defined the high and low vaccine coverage stratas post hoc according the highest and lowest quintile of coverage; cohorts with vaccine coverage up to the 20th percentile of the population (≤12% coverage) were defined as having low vaccine coverage, and those with vaccine coverage at or above the 80th percentile of the population (≥30% coverage) were defined as having high vaccine coverage. Ideally, VE analysis might have used a group receiving placebo as the appropriate control group, but for this dataset, there was no such ideal group. Thus, the cohorts in the lowest quintile of vaccine coverage served as the best control. For the primary analysis, we used the time period of the first 2 y after vaccination, but also calculated VE for longer time periods. Since transmission risk might relate to the age of the index case, we also evaluated VE for cohorts where the index cases were <5 y and cohorts where the index case was ≥5 y old.

A multivariable log-binomial regression model was used to calculate relative risk (with 95% confidence intervals) between the high vaccine coverage cohorts and low vaccine coverage cohorts as previously defined. The relative risk was then transformed into VE as (1 − relative risk) × 100%. We also used the Firth penalized likelihood method, an approach that reduces small-sample bias in maximum likelihood estimation [13], to evaluate the sensitivity of the estimates using the log-binomial model.

We estimated the overall and indirect VE of the vaccine. The overall VE was assessed by comparing the attack rates between high and low vaccine coverage cohorts exposed to cholera index cases irrespective of the vaccination status of the individuals in the cohorts. The indirect VE was assessed by comparing the attack rates among unvaccinated individuals between high and low vaccine coverage cohorts exposed to cholera index cases.

Since the high and low vaccine coverage cohorts were defined post hoc, we also evaluated VE against non-cholera diarrhea, defined as watery diarrhea (no blood in stools) and a fecal culture negative for *Vibrio cholerae* O1, in a bias indicator analysis. This bias indicator analysis was designed to assess whether the results with respect to VE against cholera could be attributed to bias [14]. An absence of VE in the bias indicator analysis would be interpreted as suggesting an absence of bias in the VE against cholera. We used SAS version 9.3 for the data analysis.

## Results

### Study Population

**Fig 1:**
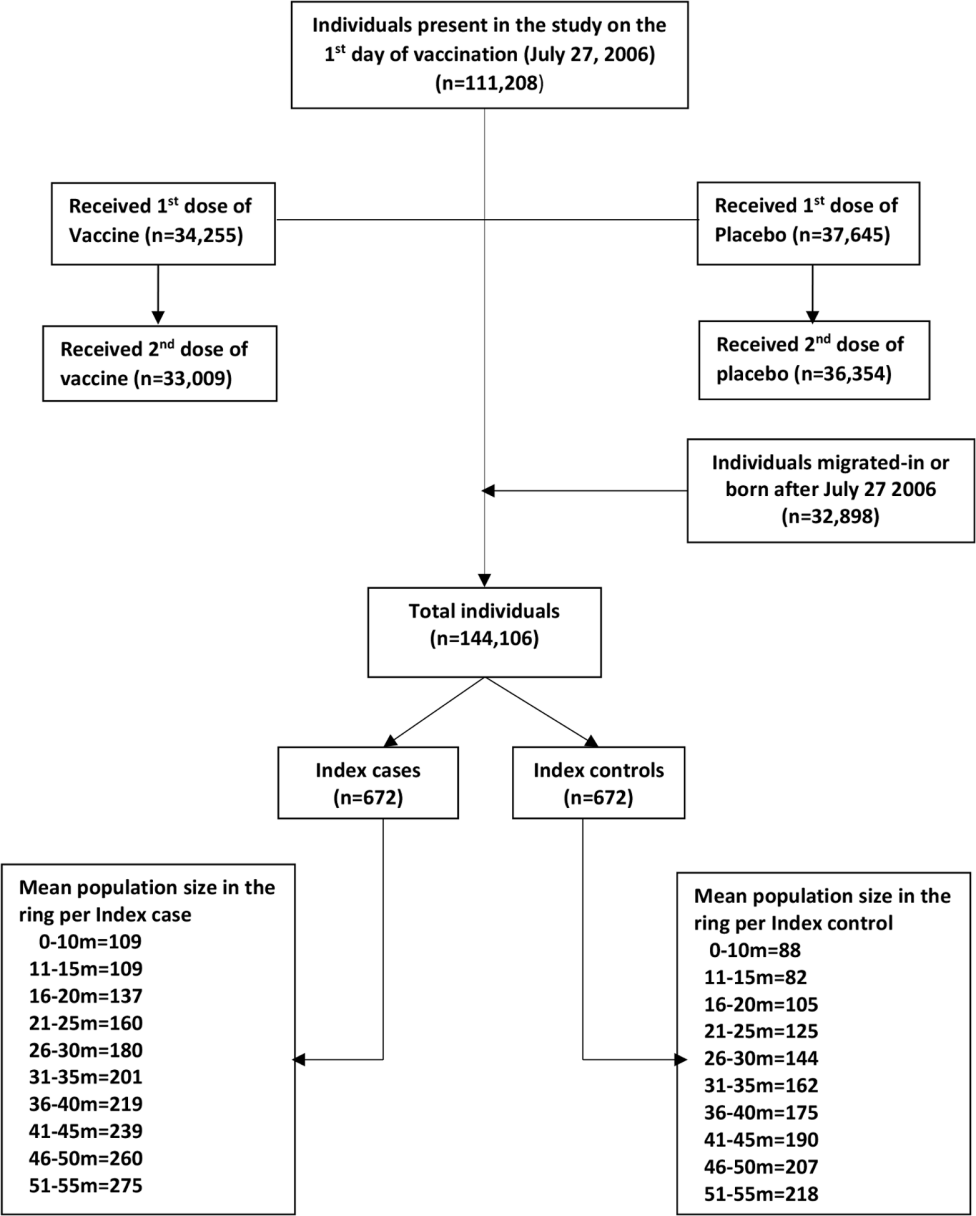
CONSORT flow chart for assembling the population. The ring 0–10 m includes individuals living 0.00 m to 10.00 m from the index case/control, the ring 11–15 m includes individuals living 10.01 m to 15.00 m from the index case/control, and so on.

There were 111,208 individuals present in the study area on the first day of vaccination (27 July 2006). A total of 32,898 individuals migrated into or were born in the study area during the 5-y follow-up period, totaling 144,106 individuals under surveillance. Among these individuals, 71,900 participated in the vaccine trial and received at least one dose of either vaccine or placebo; 33,009 individuals received two doses of the vaccine, and 36,354 individuals received two doses of placebo (Fig 1). The last day of vaccination was 10 September 2006. This study included cholera cases reporting in a project clinic/hospital from 1 October 2006 to until the end of the surveillance (25 September 2011).

We observed 672 episodes of cholera during the 5-y surveillance period. Accordingly, we selected 672 age-group-matched controls. We defined the exposed and non-exposed cohorts for each concentric ring centered on the location of each index case and control (see above). In a 10-m ring, we observed an average of 109 people in a case cohort and 88 people in a control cohort (Fig 1), yielding a total of 73,377 people for the case cohorts and a total of 59,334 people for the control cohorts at the 672 time points during the 5-y follow-up time. The number of cholera cases (excluding index cases), population, and incidence rate/1,000/week in the different rings are shown in S1 Table.

### Spatiotemporal Risk of Cholera

**Fig 2:**
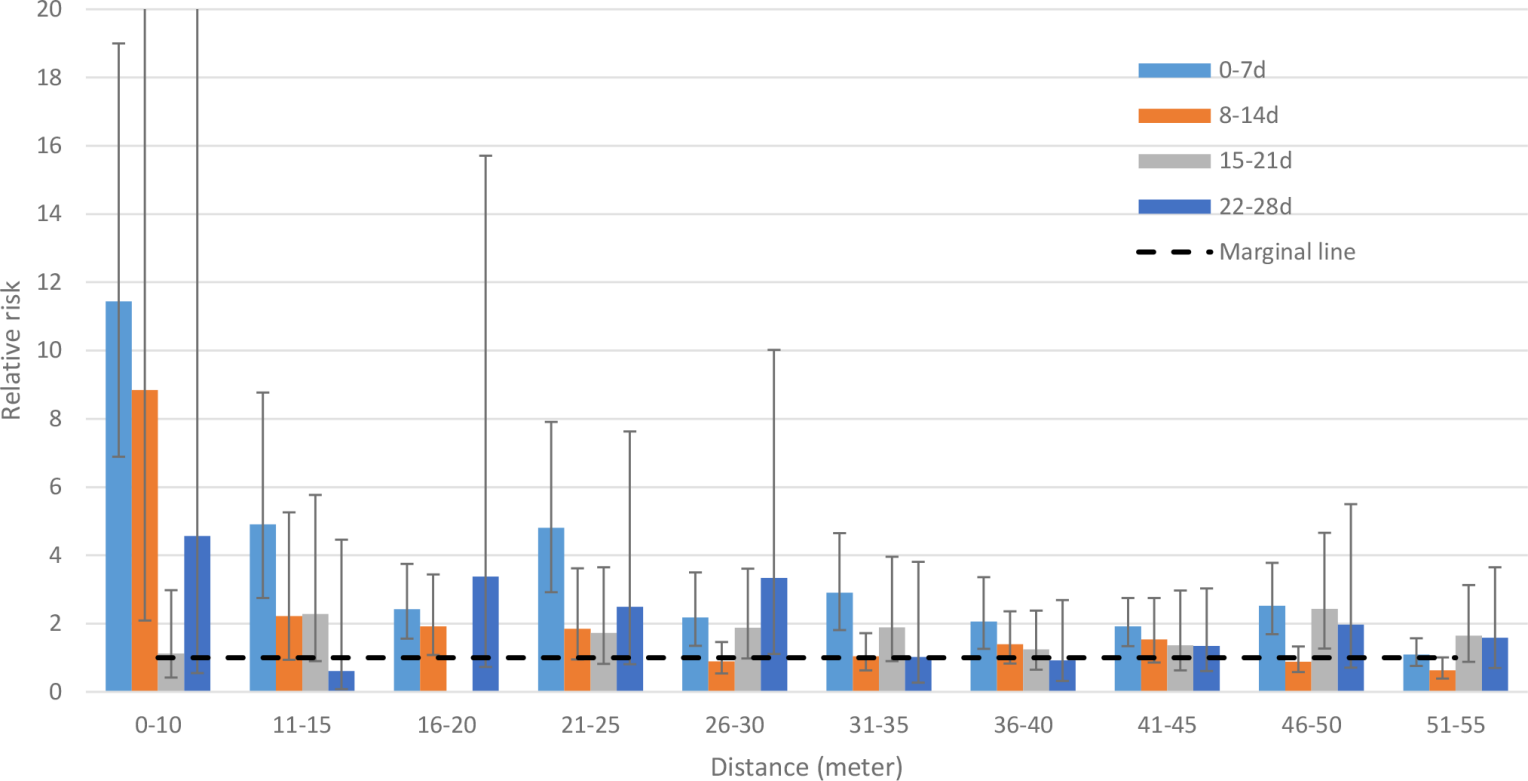
Relative risk for cholera between cohorts of cases and cohorts of controls in spatiotemporal scales. Since there were no cholera cases among cohorts of index controls in the 16–20-m ring during the 15–21-d time frame, the relative risk could not be calculated. The 95% confidence intervals are shown by error bars. We cut off the upper 95% CI of the 0–10-m ring and 8–14-d time frame (which is 37.36) for better visualization of all bars and their 95% CIs.

The risk for cholera was more than 11 times higher (95% CI 7–19) for exposed than for non-exposed cohort members when evaluating the risk within 10 m and within 7 d from the onset of illness of the index case (Fig 2; S1 Table). The risk remained significantly higher for rings up to 50 m within the first 7 d after disease onset of the index case. When we evaluated the risk using a 11–15-m ring, the risk was marginally significant until 3 wk. The risk remained elevated for 2 wk for the exposed cohorts using the 16–20-m ring. For cohorts created using a 21–25-m ring, the elevated risk remained marginally significant for 2 wk after the onset of illness of the index case. For cohorts at greater distances (>25 m) at times greater than a week, the increased risk was inconsistent (S1 Table).

The primary analysis used a ring size of 0–25 m, with a ring size of 0–50 m to assess sensitivity. The cholera risk in cohorts in rings of 0–25 m around index cases was 5-fold higher (95% CI 4–7) than that in cohorts of index controls within a week of onset of illness of their index case, and the risk was significantly elevated up to 28 d (Table 1). When analyzing the risk for cohorts living within 50 m of index cases, the risk was significantly elevated up to 35 d from the onset of the index case (S2 Table).

**Table 1:** Incidence rate and risk among individuals living within 25 m of index cases relative to individuals living within 25 m of index controls.

Time Frame	Index Cases			Index Controls			Unadjusted Estimates		Adjusted Estimates^‡^	
	Population*	Cases^†^	Incidence Rate/1,000	Population*	Cases^†^	Incidence Rate/1,000	Relative Risk	95% CI	Relative Risk	95% CI
0–7 d	345,512	548	1.59	269,058	73	0.27	5.85	4.58–7.47	5.39	4.22–6.88
8–14 d	345,512	120	0.35	269,058	37	0.14	2.53	1.75–3.65	2.35	1.62–3.40
15–21 d	345,512	68	0.20	269,058	23	0.09	2.3	1.43–3.69	2.25	1.40–3.62
22–28 d	345,512	31	0.09	269,058	9	0.03	2.68	1.28–5.63	2.47	1.17–5.21
29–35 d	345,512	33	0.10	269,058	14	0.05	1.84	0.98–3.43	1.51	0.80–2.84
36–42 d	345,512	23	0.07	269,058	11	0.04	1.63	0.79–3.34	1.39	0.67–2.86

*Cumulative total population around 672 index cases/index controls (excluding index cases).

^†^Cumulative number of cases around 672 index cases/index controls (excluding index cases).

^**‡**^Adjusted for age, sex, vaccination status, and distance to the nearest water body.

### Vaccine Effectiveness Estimates

Since the magnitude of risk was significantly elevated among cohorts living within 0–25 m of a case between 8 and 28 d, we used this spatiotemporal scale as the primary analysis when estimating VE. For the entire set of cohorts living within 25 m of index cases, the median vaccine coverage was 22%. The median coverage in the high coverage rings (coverage ≥ 30%) was 41% (interquartile range 34%–44%), compared to 0% (interquartile range 0%–6%) in the low vaccine coverage rings (coverage ≤ 12%). During the first 2 y, the overall adjusted VE was 91% (95% CI 62%–98%, *p* = 0.0011) when comparing cholera attack rates between individuals living in high vaccine coverage rings and individuals living in low vaccine coverage rings (Table 2). Using data from a 3-y surveillance period, the overall adjusted VE was 75% (95% CI 44%–89%, *p* < 0.001) among individuals living in higher vaccine coverage rings. Overall, the adjusted VE remained significant during 5 y post-vaccination, but the magnitude of protection declined over time (Table 3).

**Table 2:** Overall and indirect vaccine effectiveness against cholera using ring vaccination strategy

Duration of Follow-Up	High Vaccine Coverage Cohorts* (Coverage ≥ 30%)		Low Vaccine Coverage Cohorts* (Coverage ≤ 12%)		VE (95% CI; *p*-Value)	
	Index Cases/Population^†^	Number of Cases^‡^ (IR/1,000)	Index Cases/Population^†^	Number of Cases^‡^ (IR/1,000)	Crude	Adjusted^£^
**Overall VE**						
2 y	55/22,344	2 (0.09)	51/21,254	22 (1.04)	91% (63 to 98; <0.001)	91% (62 to 98; 0.0011)
3 y	116/46,059	7 (0.15)	95/40,380	29 (0.72)	79% (52 to 91; <0.001)	75% (44 to 89; <0.001)
4 y	156/64,295	17 (0.26)	136/62,287	44 (0.71)	63% (35 to 79; <0.001)	62% (32 to 79; 0.0012)
5 y	182/72,978	18 (0.25)	151/68,790	44 (0.64)	62% (33 to 78; <0.001)	62% (31 to 79; <0.001)
**Indirect VE**						
2 y	55/12,998	1 (0.08)	51/20,624	22 (1.07)	93% (47 to 99; 0.0101)	93% (44 to 99; 0.0113)
3 y	116/27,452	5 (0.18)	95/38,615	29 (0.75)	76% (37 to 91; 0.0034)	72% (27 to 89; 0.0088)
4 y	156/38,760	13 (0.34)	136/59,207	44 (0.74)	55% (16 to 76; 0.0117)	52% (10 to 75; 0.0233)
5 y	182/43,900	14 (0.32)	151/65,299	44 (0.67)	53% (14 to 74; 0.0147)	51% (8 to 74; 0.0257)

*The vaccine coverage of individuals living within 25 m of index cases was calculated as the number of two-dose vaccine recipients divided by all population within 25 m.

^†^Number of index cases over cumulative total population within 25 m of the index cases.

^**‡**^Cumulative total cholera cases within 25 m of the index cases (excluding index cases) and within 8–28 d of onset of index cases.

^£^Adjusted for age for the 2-y analysis, for age and sex for the 3-y analysis, and for age, sex, and distance from household to nearest water body for the 4-y and 5-y analyses.

IR, incidence rate; VE, vaccine effectiveness.

**Table 3:** Overall vaccine effectiveness against cholera using ring vaccination strategy, by age group of the index case.

Duration of Follow-Up	High Vaccine Coverage Cohorts* (Coverage ≥ 30%)		Low Vaccine Coverage Cohorts* (Coverage ≤ 12%)		Vaccine Effectiveness (95% CI; *p*-Value)	
	Population^†^	Number of Cases^‡^	Population^†^	Number of Cases^‡^	Crude	Adjusted^£^
**Age group: <5 y**						
2 y	1,752	0	1,848	12	100%	**
3 y	3,607	2	3,396	15	88% (45 to 97; 0.0058)	86% (39 to 97; 0.0095)
4 y	4,978	7	5,166	18	60% (4 to 83; 0.0414)	56% (−9 to 82; 0.0776)
5 y	5,611	7	5,649	18	61% (4 to 84; 0.0351)	57% (−7 to 83; 0.0684)
**Age group: ≥5 y**						
2 y	20,592	2	19,406	10	81% (14 to 96; 0.0312)	**
3 y	42,452	5	36,984	14	69% (14 to 89; 0.0250)	66% (5 to 88; 0.0402)
4 y	59,317	10	57,121	26	63% (23 to 88; 0.0076)	66% (27 to 84; 0.0059)
5 y	67,367	11	63,141	26	60% (20 to 80; 0.0101)	65% (26 to 84; 0.0064)

*The vaccine coverage of individuals living within 25 m of index cases was calculated as the number of two-dose vaccine recipients divided by all population within 25 m.

^†^Cumulative total population within 25 m of the index cases.

^**‡**^Cumulative total cholera cases within 25 m of the index cases (excluding index cases) and within 8–28 d of onset of index cases.

^£^Adjusted for distance from household to nearest water body.

**Not enough cases to develop a multivariable model.

When evaluating rates of cholera among individuals who did not receive vaccine, the indirect adjusted VE among individuals (for the period 8 to 28 d) living in a high vaccine coverage ring was 93% (95% CI 44%–99%, *p* = 0.01) for the first 2 y after vaccination (Table 2). As with overall VE, the magnitude of indirect VE gradually decreased over time but remained significant for 5 y. Indirect VE was 51% (95% CI 8%–74%, *p* = 0.02) during 5 y of surveillance (Table 3). Both overall and indirect protection was similar when the index cases were aged <5 y or ≥5 y (Tables 3 and 4). The overall VE estimates from the analysis using the Firth method were similar to the estimates obtained from the log-binomial model (S3 Table).

**Table 4:** Indirect vaccine effectiveness against cholera using ring vaccination strategy, by age group of the index case.

Duration of Follow-Up	High Vaccine Coverage Cohorts* (Coverage ≥ 30%)		Low Vaccine Coverage Cohorts* (Coverage ≤ 12%)		Vaccine Effectiveness (95% CI; *p*-Value)	
	Population^†^	Number of Cases^‡^	Population^†^	Number of Cases^‡^	Crude	Adjusted^£^
**Age group: <5 y**						
2 y	1,261	0	1,814	12	100%	**
3 y	2,985	2	3,345	15	85% (35 to 97; 0.0114)	84% (27 to 96; 0.0175)
4 y	4,332	7	5,114	18	54% (−9 to 81; 0.0801)	50% (−24 to 80; 0.1380)
5 y	4,965	7	5,597	18	66% (−5 to 82; 0.0638)	52% (−19 to 80; 0.1153)
**Age group: ≥5 y**						
2 y	11,737	1	18,810	10	84% (−25 to 98; 0.0808)	**
3 y	24,467	3	35,270	14	69% (−7 to 91; 0.0649)	**
4 y	34,428	6	54,093	26	64% (12 to 85; 0.0251)	62% (6 to 85; 0.0361)
5 y	38,935	7	59,702	26	59% (5 to 82; 0.0377)	59% (1 to 83; 0.0469)

*The vaccine coverage of individuals living within 25 m of index cases was calculated as the number of two-dose vaccine recipients divided by all population within 25 m.

^†^Cumulative total population within 25 m of the index cases.

^**‡**^Cumulative total cholera cases within 25 m of the index cases (excluding index cases) and within 8–28 d of onset of index cases.

^£^Adjusted for distance from household to nearest water body for the 3-y analysis, and for age and distance from household to nearest water body for the 4-y and 5-y analyses.

**Not enough cases to develop a multivariable model.

The bias indicator analysis evaluating overall and indirect VE against non-cholera diarrhea demonstrated no protection of the vaccine against non-cholera diarrhea in any of the surveillance years (Table 5). However, we observed a significantly higher non-cholera diarrhea attack rate among individuals living in the high vaccine coverage rings than among individuals living in the low vaccine coverage rings for the 4- and 5-y surveillance periods.

**Table 5:** Overall and indirect vaccine effectiveness against non-cholera diarrhea using ring vaccination strategy.

Duration of Follow-Up	High Vaccine Coverage Cohorts* (Coverage ≥ 30%)		Low Vaccine Coverage Cohorts* (Coverage ≤ 12%)		VE (95% CI; *p*-Value)	
	Index Cases/Population^†^	Number of Cases^‡^ (IR/1,000)	Index Cases/Population^†^	Number of Cases^‡^ (IR/1,000)	Crude	Adjusted^£^
**Overall VE**						
2 y	55/22,344	139 (6.22)	51/21,254	154 (7.25)	14% (−8 to 32; 0.1909)	10% (−14 to 29; 0.3737)
3 y	116/46,059	341 (7.40)	95/40,380	295 (7.31)	−1% (−18 to 13; 0.8665)	−4% (−22 to 12; 0.6599)
4 y	156/64,295	524 (8.15)	136/62,287	453 (7.27)	−12% (−27 to 1; 0.0748)	−14% (−29 to 0; 0.0508)
5 y	182/72,978	600 (8.22)	151/68,790	496 (7.21)	−14% (−29 to −1; 0.1962)	−16% (−32 to −3; 0.0149)
**Indirect VE**						
2 y	55/12,998	79 (6.08)	51/20,624	149 (7.22)	16% (−10 to 36; 0.2128)	10% (−18 to 32; 0.4454)
3 y	116/27,452	228 (8.31)	95/38,615	281 (7.28)	−14% (−36 to 4; 0.1365)	−16% (−39 to 3; 0.1052)
4 y	156/38,760	362 (9.34)	136/59,207	428 (7.23)	−29% (−49 to −12; <0.001)	−30% (−51 to −13; <0.001)
5 y	182/43,900	405 (9.23)	151/65,299	470 (7.20)	−28% (−47 to −12; <0.001)	−29% (−49 to −12; <0.001)

*The vaccine coverage of individuals living within 25 m of index cases was calculated as the number of two-dose vaccine recipients divided by all population within 25 m.

^†^Number of index cases over/cumulative total population within 25 m of the index cases.

^**‡**^Cumulative total non-cholera diarrhea cases within 25 m of the index cases (excluding index cases) and within 8–28 d of the onset of index case.

^£^Adjusted for age, sex, and distance to nearest water body.

IR, incidence rate; VE, vaccine effectiveness.

We conducted a sensitivity analysis using cohorts living within 50 m of index cases. Since the increased risk remained significant up to 35 d in these cohorts, we included cases occurring between 8 and 35 d after the index case. For this analysis, we defined high vaccine coverage as ≥33% to have a similar sized population as the low vaccine coverage cohorts during the first 2 y of surveillance. There was no notable difference in overall or indirect VE among these 50-m ring cohorts (S4 Table) compared to the cohorts of 25-m rings (Table 2). Both the overall and indirect VE were similar when index cases were children aged <5 y or were older (S5 and S6 Tables). Similar to the analysis for the cohorts of 25-m rings, the bias indicator analysis of the cohorts of 50-m rings did not show any overall or indirect VE against non-cholera diarrhea in any of the surveillance years except a slightly higher VE in the fifth year of surveillance (S7 Table).

## Discussion

This retrospective analysis of cholera vaccine trial data from an endemic setting provides an opportunity to evaluate the dynamics of cholera transmission and OCV effectiveness among contacts of cases, as a surrogate for a ring vaccination strategy. The results demonstrate that the risk for cholera among individuals living within 10 m of a cholera case and within 2 wk from the onset of cholera for the index case is extremely high (9- to 11-fold) compared to the risk among individuals living within the same distance of a control (non-cholera case) within same time frame. Such an elevated risk in this defined distance and time frame around a case strongly suggests transmission of cholera among persons living in the same household or close by [15,16]. The higher risk for cholera among individuals living as far as 25 m from a cholera case in this highly populated urban slum setting illustrates that transmission extends beyond the immediate household and supports the need for interventions targeted to individuals living outside the immediate household.

The higher risk for cholera among individuals living near a cholera case also depends on the time since the onset of illness of the case. Our results show the risk to be extremely high within 7 d of the onset of illness but that the increased risk decreases over the next 28 d, after which it is not significant. This brief high-risk interval indicates the need to intervene very quickly if one hopes to intervene in the transmission of the disease.

To assess the potential benefit of a ring vaccination strategy, we evaluated VE among people living within 25 m of cholera cases between 8 d and 28 d of the onset of illness of the case. We used the time frame 8–28 d based on the assumption that if individuals were vaccinated in a ring vaccination strategy, protection would start 4–7 d following vaccination [17,18] and that the increased risk of cholera would not be sustained after 28 d from the time of the case. In this study, individuals living in high vaccine coverage rings (≥30% coverage) within 25 m of a case, regardless of vaccination status, were highly protected (VE of 91%) compared to a individuals living in low vaccine coverage rings (≤12% coverage). Even individuals who had not received vaccine in the cholera vaccine trial were similarly protected if they were residing in a high coverage ring. Notably, significant VE continued for 5 y after receiving the vaccine, suggesting that revaccination may not be needed if another case is observed in the same community within 5 y, assuming that vaccine coverage is high.

Our analysis suggests that a ring vaccine strategy may be highly effective if ≥30% coverage can be quickly achieved. Delivering vaccine quickly (within a day or two) to neighbors of cholera cases will be challenging, but may be possible in some areas. Such a strategy assumes that the short-term protection seen with two doses will begin with the first dose and that a second follow-up dose can also be administered soon after (about 2 wk). In fact, there is evidence of protection from a single dose [19,20].

Since cholera transmission and VE might be affected by the age of the index case, we evaluated whether VE was altered by the age group of the index case. In this analysis, age of the index case did not change VE.

To demonstrate that these results are not affected by bias resulting from the cohorts being defined post hoc without randomization, we conducted a bias indicator analysis. This evaluation found no protection against non-cholera diarrhea by the vaccine, thus supporting the notion that our study was not biased by an ecological effect. The higher non-cholera diarrhea attack rate among individuals living in higher vaccine coverage rings compared to the rate among individuals living in lower vaccine coverage rings could be related to differences in healthcare-seeking behavior between these two populations, as several studies have observed a lower turn-out rate among non-vaccinated individuals in hospitals/clinics [21,22].

Our results are conservative since the threshold for defining higher vaccine coverage was 30%, i.e., only 30% of a cohort had to be vaccinated for it to be considered a high coverage cohort. Second, the low vaccination coverage cohorts were not a completely unvaccinated population. The findings of an overall VE of 91% with vaccine coverage of ≥30% is similar to a simulation study conducted by Longini and colleagues [6]. Note that the simulation study assumed 0% coverage for the control group, but coverage was ≤12% in the comparison group used in our study. Our study was not sufficiently large to compare cohorts with 0% coverage with others with 50% coverage, such that our results could be directly compared with the simulation study. However, our results do suggest that cholera transmission could be nearly eliminated by achieving at least 50% coverage around cases.

The major strength of our study is high-quality data collected during this randomized controlled trial, which was conducted in an ideal setting. The community was mobilized throughout the surveillance period, and the residents of the community were encouraged to seek care at a project clinic/hospital in the event of diarrhea. High-quality laboratory procedures were established for fecal microbiology, and intensive data monitoring insured a completely error-free dataset.

Although the data quality is high, there are several limitations of this analysis. First, regarding the definition of high and low vaccine coverage cohorts, a better analysis might have compared cohorts with 0% coverage with cohorts with >50% coverage. However, this type of analysis would have required a much larger database. Second, the study was conducted in a densely populated slum area of Kolkata, an area with endemic cholera, and many people may have been naturally exposed to cholera earlier. This area is not representative of all areas at risk for cholera, especially rural areas without previous exposure. Although the concepts identified in this study may relate to other areas at risk, it would seem that one would have to consider much larger sized rings in areas with less dense populations. Since there were about 500 people in the 25-m rings identified as highest risk, a population size of 500 to 1,000 may be an approximate population number to consider when conducting such analyses in other areas.

There are also limitations to using the data from the cholera vaccine trial to simulate the benefits of a ring vaccination strategy. The original campaign delivered two doses of vaccine to participants over a short time and then followed them prospectively for 5 y. This analysis then attempts to use these data to estimate the benefits of achieving high coverage among cohorts close to cases using a ring vaccination strategy. In practice, ring vaccination campaigns in which 500–1,000 neighbors are given vaccine will be challenging, and protection would have to depend on protection derived from herd protection starting with the first dose, even though the second dose could be given later. Such a ring vaccination strategy would depend on vaccine being available on site, with field teams trained and ready to vaccinate immediately when a case is confirmed. It would also depend on clinical teams screening patients for cholera and laboratory staff being able to carry out rapid testing of fecal specimens. The clinical, laboratory, and field teams would need to be well coordinated, thus adding to the difficulty of such a strategy.

A second limitation of the hypothetical ring vaccination strategy represented by this study is the several days’ delay until the development of protective immunity; thus, other strategies would be needed to prevent cases during the first week after diagnosis of the index case, which is the time of highest risk. An intensive WASH (water, sanitation, and hygiene) strategy was found to be effective in reducing infections in households of cases and may be part of a comprehensive intervention strategy [23]. Though an immune response will take a few days, in a cholera endemic area like Kolkata, a single dose might induce a more rapid booster response, thus initiating protection more quickly than in cholera naïve areas. In such an area, a single dose would have to be protective, as was recently observed in Dhaka, Bangladesh [20].

Policy makers will need to select OCV strategies most likely to have an impact on transmission while maximizing the cost-effectiveness of OCV use [24]. Transmission hotspots of diseases have recently been exploited to devise novel prevention and control approaches [25,26]. Since the risk is extremely high among people living close to a case, these may be considered “transmission hotspots.” Our study suggests that a ring vaccination strategy could effectively reduce the threat of cholera by breaking the transmission chain. Since this strategy targets only the households around the case, and not the entire locality, the number of doses required is much smaller, potentially improving the cost-effectiveness of vaccination substantially.

In conclusion, our results illustrate the high risk to individuals living close to cholera cases and provide evidence that ring vaccination could be an effective strategy to consider when controlling cholera in cholera-affected areas. Further studies are needed to test the feasibility and effectiveness of ring vaccination, and to determine how to integrate vaccination into a more comprehensive and integrated set of interventions to prevent cases and prevent transmission.

## Supporting Information

S1 Table Unadjusted and adjusted estimates of relative risk at different spatiotemporal scales. (XLSX)

S2 Table Incidence rate and risk among individuals living within 50 m of index cases relative to individuals living within 50 m of index controls. (DOCX)

S3 Table Overall vaccine effectiveness against cholera using ring vaccination strategy using Firth penalized likelihood method. (DOCX)

S4 Table Overall and indirect vaccine effectiveness against cholera using ring vaccination strategy. (DOCX)

S5 Table Overall vaccine effectiveness against cholera among cohorts of the index cases aged <5 y and among cohorts of index cases aged ≥5 y using ring vaccination strategy. (DOCX)

S6 Table Indirect vaccine effectiveness against cholera among cohorts of index cases aged <5 y and among cohorts of index cases aged ≥5 y using ring vaccination strategy. (DOCX)

S7 Table Overall and indirect vaccine effectiveness against non-cholera diarrhea using ring vaccination strategy. (DOCX)

S1 Text STROBE checklist. (PDF)

S2 Text Analysis plan. (PDF)

## Abbreviations

OCV: oral cholera vaccine
VE: vaccine effectiveness
