# Supplementary material for: Potential for Controlling Cholera Using a Ring Vaccination Strategy: Re-analysis of Data from a Cluster-Randomized Clinical Trial
Source: PLoS Med. 2016 Sep 13;13(9):e1002120. doi: 10.1371/journal.pmed.1002120 (PMC5021260; doi:10.1371/journal.pmed.1002120)
Supplement: S2 Table — (DOCX) [file pmed.1002120.s002.docx]

Table S2. Incidence rate and risk among cohorts of index cases living within 50 meters of the index cases relative to among cohorts of index controls living within 50 meters of the index controls

| Time period | Index cases | | | Index controls | | | Unadjusted estimates | | Adjusted  Estimates**^‡^** | |
| --- | --- | --- | --- | --- | --- | --- | --- | --- | --- | --- |
|  | Population* | Cases^†^ | Incidence rate/100,0000 | Population* | Cases* | Incidence rate/100,0000 | Relative risk | 95% CI | Relative  risk | 95% CI |
| 0-7 days | 1,084,483 | 978 | 90.18 | 859,058 | 212 | 24.68 | 3.66 | 3.15-4.24 | 3.39 | 2.92-3.93 |
| 8-14 days | 1,084,483 | 314 | 28.95 | 859,058 | 176 | 20.49 | 1.41 | 1.17-1.70 | 1.35 | 1.12-1.62 |
| 15-21 days | 1,084,483 | 202 | 18.63 | 859,058 | 83 | 9.66 | 1.93 | 1.49-2.49 | 1.89 | 1.46-2.44 |
| 22-28 days | 1,084,483 | 92 | 8.48 | 859,058 | 37 | 4.31 | 1.97 | 1.34-2.88 | 1.81 | 1.23-2.65 |
| 29-35 days | 1,084,483 | 77 | 7.10 | 859,058 | 26 | 3.03 | 2.35 | 1.50-3.66 | 1.95 | 1.25-3.05 |
| 36-42 days | 1,084,483 | 48 | 4.43 | 859,058 | 25 | 2.91 | 1.52 | 0.94-2.46 | 1.37 | 0.84-2.22 |

*Cumulative total population of the 672 cohorts of index cases/index controls (excluding index cases)

^†^Cumulative number of cases among population of the 672 cohorts of index cases/index controls (excluding index cases/controls)
**^‡^**Adjusted for age, sex, vaccination status, and distance from water bodies
