# Supplementary material for: Potential for Controlling Cholera Using a Ring Vaccination Strategy: Re-analysis of Data from a Cluster-Randomized Clinical Trial
Source: PLoS Med. 2016 Sep 13;13(9):e1002120. doi: 10.1371/journal.pmed.1002120 (PMC5021260; doi:10.1371/journal.pmed.1002120)
Supplement: S3 Table — (DOCX) [file pmed.1002120.s003.docx]

Table S3. Overall vaccine effectiveness against cholera using ring vaccination strategy using Firth penalized likelihood method

| Duration of follow-up | High vaccine coverage cohorts*  (coverage≥30%) | | Low vaccine coverage cohorts*  (coverage≤12%) | | Vaccine effectiveness (%)  (95% CI; p-value) | |
| --- | --- | --- | --- | --- | --- | --- |
|  | Index cases/  Population† | No. of  cases‡ (IR/1000) | Index cases/  Population† | No. of cases‡ (IR/1000) | Crude | Adjusted£ |
| Years 1-2 | 55/22,344 | 2 (0.09) | 51/21,254 | 22 (1.04) | 89 (61 to 87; .0007) | 89 (60 to 97; .0018) |
| Years 1-3 | 116/46,059 | 7 (0.15) | 95/40,380 | 29 (0.72) | 78 (50 to 90; .0002) | 77 (49 to 90; .0003) |
| Years 1-4 | 156/64,295 | 17 (0.26) | 136/62,287 | 44 (0.71) | 62 (34 to 78; .0006) | 61 (31 to 78; .0012) |
| Years 1-5 | 182/72,978 | 18 (0.25) | 151/68,790 | 44 (0.64) | 61 (33 to 77; .0007) | 61 (31 to 78; .0012) |

*The vaccine coverage within the 25 meters around index cases was calculated by number of two-dose vaccine recipients divided by all population within 25 meters

^†^Cumulative total population within 25 meters of the index cases

**^‡^**Cumulative total cholera cases within 25 meters of the index cases (excluding index cases) and within 8-28 days of onset of index cases

^£^Adjusted for age in 1-2 year analysis, age and sex in 1-3 year analysis, and for age, sex, and distance from water bodies to household for 1-4 year and 1-5 year analyses.
