# Supplementary material for: Potential for Controlling Cholera Using a Ring Vaccination Strategy: Re-analysis of Data from a Cluster-Randomized Clinical Trial
Source: PLoS Med. 2016 Sep 13;13(9):e1002120. doi: 10.1371/journal.pmed.1002120 (PMC5021260; doi:10.1371/journal.pmed.1002120)
Supplement: S4 Table — (DOCX) [file pmed.1002120.s004.docx]

Table S4. Overall and indirect vaccine effectiveness against cholera using ring vaccination strategy

| Duration of follow-up | High vaccine coverage cohorts*  (coverage≥33%) | | Low vaccine coverage cohorts*  (coverage≤12%) | | Vaccine effectiveness (%)  (95% CI; p-value) | |
| --- | --- | --- | --- | --- | --- | --- |
|  | Index cases/  Population^†^ | No. of  cases**^‡^** (IR/1000) | Index cases/  Population^†^ | No. of cases**^‡^** (IR/1000) | Crude | Adjusted^£^ |
| **Overall vaccine effectiveness** | | | | | | |
| 1-2 year | 36/29,716 | 3 (0.10) | 31/30,469 | 42 (1.38) | 93 (76 to 98; <.0001) | 90 (66 to 97; .0002) |
| 1-3 year | 71/52,080 | 10 (0.19) | 41/42,711 | 47 (1.10) | 83 (66 to 91; <.0001) | 80 (61 to 80; <.0001) |
| 1-4 year | 81/60,600 | 16 (0.26) | 58/51,484 | 47 (0.91) | 71 (49 to 84; <.0001) | 66 (39 to 81; .0002) |
| 1-5 year | 99/72,161 | 20 (0.28) | 66/56,699 | 47 (0.83) | 64 (44 to 80; <.0001) | 58 (29 to 75; .0012) |
| **Indirect vaccine effectiveness** | | | | | | |
| 1-2 year | 36/18,048 | 2 (0.11) | 31/29,017 | 42 (1.45) | 92 (68 to 98; .0004) | 89 (56 to 97; .0020) |
| 1-3 year | 71/31,301 | 7 (0.22) | 45/40,132 | 47 (1.17) | 81 (58 to 91; <.0001) | 81 (58 to 92; <.0001) |
| 1-4 year | 81/36,465 | 10 (0.27) | 58/48,142 | 47 (0.98) | 72 (44 to 86; .0003) | 70 (41 to 85; .0005) |
| 1-5 year | 99/43,488 | 14 (0.32) | 63/52,897 | 47 (0.89) | 64 (34 to 80; .0009) | 59 (25 to 77; .0038) |

Note: There were no cases in the 4^th^ and 5^th^ year in low vaccine coverage cohorts, and all the cases were among unvaccinated population in these cohorts.

*The vaccine coverage within the 50 meters around index cases was calculated by number of two-dose vaccine recipients divided by all population within 50 meters

^†^Cumulative total population within 50 meters of the index cases

**^‡^**Cumulative total cholera cases within 50 meters of the index cases (excluding index cases) and within 8-35 days of onset of index cases

^£^Adjusted for age, sex, and distance from water bodies to household
