# Supplementary material for: Potential for Controlling Cholera Using a Ring Vaccination Strategy: Re-analysis of Data from a Cluster-Randomized Clinical Trial
Source: PLoS Med. 2016 Sep 13;13(9):e1002120. doi: 10.1371/journal.pmed.1002120 (PMC5021260; doi:10.1371/journal.pmed.1002120)
Supplement: S5 Table — (DOCX) [file pmed.1002120.s005.docx]

Table S5. Overall vaccine effectiveness against cholera among cohorts of the index cases <5 years and among cohorts of index cases ≥5 years using ring vaccination strategy

| Duration of follow-up | High vaccine coverage cohorts*  (coverage≥33%) | | Low vaccine coverage cohorts*  (coverage≤12%) | | Vaccine effectiveness (%)  (95% CI; p-value) | |
| --- | --- | --- | --- | --- | --- | --- |
|  | Population^†^ | No. of  cases**^‡^** | Index cases/  Population^†^ | No. of cases**^‡^** | Crude | Adjusted^£^ |
| **Cohorts of cases <5 years** | | | | | | |
| 1-2 year | 2057 | 0 | 2601 | 27 | 100 | ** |
| 1-3 year | 3458 | 2 | 3632 | 31 | 93 (72-98; .0002) | 93 (72-98; .0002) |
| 1-4 year | 4044 | 5 | 4254 | 31 | 83 (57-97; .0002) | 82 (55-93; .0003) |
| 1-5 year | 4734 | 5 | 4630 | 31 | 84 (60-94; .0001) | 83 (56-93; .0003) |
| **Cohorts of cases ≥5 years** | | | | | | |
| 1-2 year | 27659 | 3 | 27868 | 15 | 80 (30-94; .0113) | 77 (22-93; .0191) |
| 1-3 year | 48622 | 8 | 39079 | 16 | 60 (6-83; .0352) | 64 (16-85; .0192) |
| 1-4 year | 56556 | 11 | 47230 | 16 | 43 (-24 to 73; .16) | 44 (-22 to 74; .14) |
| 1-5 year | 67427 | 15 | 52069 | 16 | 28 (-46 to 64; .36) | 27 (-49 to 64; .39) |

*The vaccine coverage within the 50 meters around index cases was calculated by number of two-dose vaccine recipients divided by all population within 50 meters

^†^Cumulative total population within 50 meters of the index cases

**^‡^**Cumulative total cholera cases within 50 meters of the index cases (excluding index cases) and within 8-35 days of onset of index cases

^£^ Adjusted for distance from water bodies to household

** Not enough cases to develop a multivariable model
