# Supplementary material for: Potential for Controlling Cholera Using a Ring Vaccination Strategy: Re-analysis of Data from a Cluster-Randomized Clinical Trial
Source: PLoS Med. 2016 Sep 13;13(9):e1002120. doi: 10.1371/journal.pmed.1002120 (PMC5021260; doi:10.1371/journal.pmed.1002120)
Supplement: S6 Table — (DOCX) [file pmed.1002120.s006.docx]

Table S6. Indirect vaccine effectiveness against cholera among cohorts of the index cases <5 years and among cohorts of index cases ≥5 years using ring vaccination strategy

| Duration of follow-up | High vaccine coverage cohorts*  (coverage≥33%) | | Low vaccine coverage cohorts*  (coverage≤12%) | | Vaccine effectiveness (%)  (95% CI; p-value) | |
| --- | --- | --- | --- | --- | --- | --- |
|  | Population^†^ | No. of  cases**^‡^** | Index cases/  Population^†^ | No. of cases**^‡^** | Crude | Adjusted^£^ |
| **Cohorts of cases <5 years** | | | | | | |
| 1-2 year | 1502 | 0 | 2539 | 27 | 100 | ** |
| 1-3 year | 2801 | 2 | 3546 | 31 | 92 (65-92; .0006) | 92 (67-98;.0005) |
| 1-4 year | 3378 | 5 | 4185 | 31 | 80 (49-92;.0008) | 80 (48-92;.0001) |
| 1-5 year | 4068 | 5 | 4541 | 31 | 82 (54-93; .0004) | 81 (50-93;.0007) |
| **Cohorts of cases ≥ years** | | | | | | |
| 1-2 year | 16528 | 2 | 26478 | 15 | 79 (7-95; .0403) | ** |
| 1-3 year | 28500 | 5 | 36586 | 16 | 60 (-9 to 85; .0746) | 70 (15-89; .0238) |
| 1-4 year | 33087 | 5 | 43977 | 16 | 59 (-12 to 85; .0863) | 66 (1-87; .0482) |
| 1-5 year | 39420 | 9 | 48356 | 16 | 31 (-56-70; .3733) | 30 (-60-69; .40) |

*The vaccine coverage within the 50 meters around index cases was calculated by number of two-dose vaccine recipients divided by all population within 50 meters

^†^Cumulative total population within 50 meters of the index cases

**^‡^**Cumulative total cholera cases within 50 meters of the index cases (excluding index cases) and within 8-28 days of onset of index cases

^£^ Adjusted for distance from water bodies to household for 1-3y, and age and distance from water bodies to household for 1-4y and 1-5y.

** Not enough cases to develop a multivariable model
