# Supplementary material for: Potential for Controlling Cholera Using a Ring Vaccination Strategy: Re-analysis of Data from a Cluster-Randomized Clinical Trial
Source: PLoS Med. 2016 Sep 13;13(9):e1002120. doi: 10.1371/journal.pmed.1002120 (PMC5021260; doi:10.1371/journal.pmed.1002120)
Supplement: S7 Table — (DOCX) [file pmed.1002120.s007.docx]

Table S7. Overall and indirect vaccine effectiveness against non-cholera diarrhea using ring vaccination strategy

| Duration of follow-up | High vaccine coverage cohorts*  (coverage ≥33%) | | Low vaccine coverage cohorts*  (coverage ≤12%) | | Vaccine effectiveness (%)  (95% CI; p-value) | |
| --- | --- | --- | --- | --- | --- | --- |
|  | population^†^ | No. of cases**^‡^** (IR/1000) | population^†^ | No. of cases**^‡^** (IR/1000) | Crude | Adjusted^£^ |
| **Overall vaccine effectiveness** | | | | | | |
| 1-2 years | 29716 | 260/8.75 | 30469 | 317/10.40 | 0.84 (0.71-0.99; .0375) | 0.89 (0.75-1.05; .16) |
| 1-3 years | 52080 | 515/9.89 | 42711 | 420/9.83 | 1.01 (0.88-1.14; .93) | 1.08 (0.95-1.23; .25) |
| 1-4 years | 60600 | 592/9.77 | 51484 | 492/9.56 | 1.02 (0.91-1.15; .71) | 1.09 (0.96-1.23; .17) |
| 1-5 years | 72161 | 707/9.80 | 56699 | 531/9.37 | 1.05 (0.93-1.17; .43) | 1.13 (1.01-1.27; .0312) |
| **Indirect vaccine effectiveness** | | | | | | |
| 1-2 years | 18048 | 152/8.42 | 29017 | 314/10.82 | 0.78 (0.64-0.94; .0108) | 0.85 (0.69-1.03; .10) |
| 1-3 years | 31301 | 318/10.16 | 40132 | 416/10.37 | 0.98 (0.85-1.13; .79) | 1.05 (0.91-1.22; .48) |
| 1-4 years | 36465 | 375/10.05 | 48142 | 484/10.05 | 1.02 (0.89-1.17; .74) | 1.08 (0.95-1.24; .24) |
| 1-5 years | 43488 | 446/10.26 | 52897 | 521/9.85 | 1.04 (0.92-1.18; .52) | 1.12 (0.98-1.27;.08) |

*The vaccine coverage within the 50 meters around index cases was calculated by number of two-dose vaccine recipients divided by all population within 50 meters

^†^Cumulative total population within 50 meters of the index cases

**^‡^**Cumulative total non-cholera diarrhea cases within 50 meters of the index cases (excluding index cases) and within 8-35 days of the onset of index case

^£^Adjusted for age, sex, distance from water bodies
