# Supplementary material for: Potential for Controlling Cholera Using a Ring Vaccination Strategy: Re-analysis of Data from a Cluster-Randomized Clinical Trial
Source: PLoS Med. 2016 Sep 13;13(9):e1002120. doi: 10.1371/journal.pmed.1002120 (PMC5021260; doi:10.1371/journal.pmed.1002120)
Supplement: S1 Text — (PDF) [file pmed.1002120.s008.pdf]

STROBE Statement—checklist of items that should be included in reports of observational studies

| | Item No | Recommendation |
|---|---|---|
| **Title and abstract** | 1 | (*a*) Indicate the study's design with a commonly used term in the title or the abstract<br>We include in the subtitle "an observational study" |
| | | (*b*) Provide in the abstract an informative and balanced summary of what was done and what was found<br>The abstract contains the sections Methods, Results and Conclusion, where we summarize the results of the paper. |
| **Introduction** | | |
| Background/rationale | 2 | Explain the scientific background and rationale for the investigation being reported<br>The first three paragraphs of the Background section provide the scientific background and rationale for the investigation. |
| Objectives | 3 | State specific objectives, including any prespecified hypotheses<br>At the end of the Introduction we state that "We explored the potential of a ring vaccination strategy in controlling cholera by leveraging the data from a large clinical trial. We first explored the magnitude of risk around cholera cases at different spatio-temporal scales. Based on this exploration, we identified the suitable scale for the ring vaccination in that setting, and estimated overall and indirect protective effectiveness (PE) of OCV using the ring vaccination strategy". |
| **Methods** | | |
| Study design | 4 | Present key elements of study design early in the paper<br>Key elements of the design are included early in the Methods section. |
| Setting | 5 | Describe the setting, locations, and relevant dates, including periods of recruitment, exposure, follow-up, and data collection<br>This is addressed in subsection "The study data" |
| Participants | 6 | (*a*) *Cohort study*—Give the eligibility criteria, and the sources and methods of selection of participants. Describe methods of follow-up<br>In this study we followed the contacts of index cases and contacts of index controls (age-matched) over a period of time for evaluating risk for cholera at spatio-temporal scale, and followed the contacts of index cases living in high and low coverage rings for evaluating effectiveness of the ring vaccination strategy. Eligibility criteria as well as sources and methods for selecting the index cases and controls are discussed in subsection "The study data".<br>The rationale behind selecting age-matched controls is that the risk of transmission for cholera may vary by age of the case.<br>*Case-control study*—Give the eligibility criteria, and the sources and methods of case ascertainment and control selection. Give the rationale for the choice of cases and controls<br>*Cross-sectional study*—Give the eligibility criteria, and the sources and methods of selection of participants |
| | | (*b*) *Cohort study*—For matched studies, give matching criteria and number of exposed and unexposed<br>The matching (age group at date of admission) and the exposure criteria are defined in the subsection "The study data"<br>*Case-control study*—For matched studies, give matching criteria and the number of controls per case<br>. |

| | | |
|---|---|---|
| Variables | 7 | Clearly define all outcomes, exposures, predictors, potential confounders, and effect modifiers. Give diagnostic criteria, if applicable<br>In section "Analytical methods", we explain that the outcome (episode of cholera (yes/no) was modeled using a multivariable logistic regression to calculate relative risk (RR) and 95% confidence intervals for the relative risk between contacts living in high vaccine coverage rings versus contacts living in low vaccine coverage rings. Potential confounders are provided at the bottom of each table, and the diagnostic criteria for identifying cholera is recorded in the Analysis Plan. |
| Data sources/ measurement | 8* | For each variable of interest, give sources of data and details of methods of assessment (measurement). Describe comparability of assessment methods if there is more than one group<br>We describe the data sources in the subsection "The study data". |
| Bias | 9 | Describe any efforts to address potential sources of bias<br>We conducted a bias indicator study, which is provided in the subsection "Analytical method" |
| Study size | 10 | Explain how the study size was arrived at<br>We accumulated cases and individuals within specified distance and time around index cases and index controls over the five year time. |
| Quantitative variables | 11 | Explain how quantitative variables were handled in the analyses. If applicable, describe which groupings were chosen and why<br>Level of vaccine coverage and distance to nearest waterbody were dimensional variable, vaccination status was binary, and age was categorized into two groups: <5 years and 5 years and above. This particular age grouping was done for estimating vaccine effectiveness based on the results of the analysis in earlier work with this data set. This is define in the "Analytical methods" section. |
| Statistical methods | 12 | (*a*) Describe all statistical methods, including those used to control for confounding<br>Statistical methods are described in the "Analytical methods" section. Confounding was controlled by using a multivariable logistic regression model. |
| | | (*b*) Describe any methods used to examine subgroups and interactions<br>NA |
| | | (*c*) Explain how missing data were addressed<br>NA |
| | | (*d*) *Cohort study*—If applicable, explain how loss to follow-up was addressed<br>We included only those persons who were present in the ring during the period of observation.<br>*Case-control study*—If applicable, explain how matching of cases and controls was addressed<br>*Cross-sectional study*—If applicable, describe analytical methods taking account of sampling strategy |
| | | (*e*) Describe any sensitivity analyses<br>We did not do any sensitivity analysis in this study. Instead, we performed a bias indicator study as stated above. |

Continued on next page

| **Results** | | |
|---|---|---|
| Participants | 13* | (a) Report numbers of individuals at each stage of study—eg numbers potentially eligible, examined for eligibility, confirmed eligible, included in the study, completing follow-up, and analysed<br>This is done in the first paragraph of the Results section. However, the detailed information on the individuals analyzed in this study are given in the Supplementary documents. |
| | | (b) Give reasons for non-participation at each stage<br>NA |
| | | (c) Consider use of a flow diagram<br>Figure 1 contains the flow diagram |
| Descriptive data | 14* | (a) Give characteristics of study participants (eg demographic, clinical, social) and information on exposures and potential confounders<br>The characteristics of the study population are, published in previous studies and the references are provided. |
| | | (b) Indicate number of participants with missing data for each variable of interest<br>NA |
| | | (c) *Cohort study*—Summarise follow-up time (eg, average and total amount)<br>This is given in the supplementary document and in the footnotes of each table. |
| Outcome data | 15* | *Cohort study*—Report numbers of outcome events or summary measures over time<br>These have been presented in Table 2 of the manuscript and in the supplementary document. |
| | | *Case-control study*—Report numbers in each exposure category, or summary measures of exposure |
| | | *Cross-sectional study*—Report numbers of outcome events or summary measures |
| Main results | 16 | (*a*) Give unadjusted estimates and, if applicable, confounder-adjusted estimates and their precision (eg, 95% confidence interval). Make clear which confounders were adjusted for and why they were included<br>These have been provided in Table 2. |
| | | (*b*) Report category boundaries when continuous variables were categorized<br>We categorized vaccine coverage in the ring as high vaccine coverage ring (coverage≥30%) and low vaccine coverage ring (coverage ≤12%) reported in the Result section. |
| | | (*c*) If relevant, consider translating estimates of relative risk into absolute risk for a meaningful time period<br>NA |
| Other analyses | 17 | Report other analyses done—eg analyses of subgroups and interactions, and sensitivity analyses<br>We conducted subgroup analysis for age, which was categorized into two groups: <5 years and 5 years and older. The results are provided in Table 3. |
| **Discussion** | | |
| Key results | 18 | Summarise key results with reference to study objectives<br>We begin the Discussion section with a short summary of the main findings of our study. |
| Limitations | 19 | Discuss limitations of the study, taking into account sources of potential bias or imprecision. Discuss both direction and magnitude of any potential bias<br>We describe the limitations in paragraph 7 of the Discussion section |
| Interpretation | 20 | Give a cautious overall interpretation of results considering objectives, limitations, multiplicity of analyses, results from similar studies, and other relevant evidence<br>At the end of the limitation sub-section we provide this information. |

| | | |
|---|---|---|
| Generalisability | 21 | Discuss the generalisability (external validity) of the study results<br>In the conclusion we state that “our results provide evidence that ring vaccination could be an effective strategy for controlling cholera in cholera-affected areas. Further studies are needed to test the feasibility, and effectiveness of ring vaccination, and how to integrate vaccine into a more comprehensive and integrated set of interventions to prevent cases and prevent transmission. |
| **Other information** | | |
| Funding | 22 | Give the source of funding and the role of the funders for the present study and, if applicable, for the original study on which the present article is based<br>Funding sources are acknowledged at the end of the paper. |

*Give information separately for cases and controls in case-control studies and, if applicable, for exposed and unexposed groups in cohort and cross-sectional studies.

**Note:** An Explanation and Elaboration article discusses each checklist item and gives methodological background and published examples of transparent reporting. The STROBE checklist is best used in conjunction with this article (freely available on the Web sites of PLoS Medicine at http://www.plosmedicine.org/, Annals of Internal Medicine at http://www.annals.org/, and Epidemiology at http://www.epidem.com/). Information on the STROBE Initiative is available at www.strobe-statement.org.
