# Supplementary material for: Potential for Controlling Cholera Using a Ring Vaccination Strategy: Re-analysis of Data from a Cluster-Randomized Clinical Trial
Source: PLoS Med. 2016 Sep 13;13(9):e1002120. doi: 10.1371/journal.pmed.1002120 (PMC5021260; doi:10.1371/journal.pmed.1002120)

ANALYTICAL PLAN

Controlling cholera using a ring vaccination strategy

STOP CHOLERA
Work Together. Stop Transmission. End Deaths.

Department of International Health
Johns Hopkins Bloomberg School of Public Health
615 N.Wolfe Street, Baltimore, Maryland-21225

Contents

1. Overview....2
2. Summary....3
3. Study Population....4
4. Demographic Surveillance System (DSS)....4
5. Demographic Events....5
6. Disease Surveillance....5
7. Vaccination Period....5
8. Follow-up Period....5
9. Definition of Principal Outcome Events....6
10. Other Definitions....6
11. Data Analysis....9
11.1. Evaluation of magnitude of risk around a cholera case....10
11.2. Assessment of vaccine protective effectiveness (PE)....10
11.3. This bias-indicator study....11
12. Candidate variables for inclusion as covariates in the model....12
13. Subgroup Analyses....13
14. CONSORT for assembling the study population....14

1. Overview

- Title of the study: Controlling cholera using a ring vaccination strategy

- Original protocol from which the data will be obtained:

  A Randomized Controlled Trial of the Bivalent Killed Whole Cell Oral Cholera Vaccine in Eastern Kolkata, West Bengal, India. The protocol was registered in the US clinical trials registry (http://clinicaltrial.gov/ct2/show?cond=%22Cholera%22&rank=8) which is mandated for publication of clinical trial results. The registration number is NCT 00289224.

- Investigational Product:

  Reformulated Bivalent (anti-O1, anti-O139), Killed, Whole-cell Oral Cholera Vaccine

- Indication:

  Prevention of *V. cholerae* O1 and O139 disease

- Sponsor of the original protocol:

  The Cholera Vaccine Initiative (CHOVI) and the Phase III grants - Funded by the Bill and Melinda Gates Foundation and administered by the International Vaccine Institute

  The International Vaccine Institute
  San 4-8, Nakseongdae-dong, Kwanak-gu
  Seoul, Korea 151-919
  Tel: 82-2-872-2801
  Fax: 82-2-872-2803

- Sponsor of the current study:

  The Delivering Oral Vaccine Effectively (DOVE) project. DOVE is supported by the Bill and Melinda Gates Foundation and administered through the Johns Hopkins Bloomberg School of Public Health.

- Principal Investigator of the current study

  Dr Mohammad Ali

Department of International Health
Johns Hopkins Bloomberg School of Public Health
615 N.Wolfe Street / E5543
Baltimore, Maryland 21205
410-502-3154 (office)
410-502-6733 (fax)
Email:mali25@jhu.edu

– JHU Investigators

Dr Amanda Debes, Dr. Francisco Luquero, Dr. David A Sack

– NICED Investigators

Dr. Byomkesh Manna, Dr. Suman Kanungo, Dr. Shanta Dutta, Dr. Dipika Sur, Dr. Sujit K Bhattacharya

– IVI Investigators

Ms. Deok Ryun Kim, Ms. Ju Yeon Park, Dr. Laura Digilio

– Summary: This document describes specific procedures to be followed in statistical analysis of the study entitled "Controlling cholera using a ring vaccination strategy." This document focuses on the statistical issues related to the data analysis.

2. Summary

Objectives	To explore the potential of a ring vaccination strategy in controlling cholera by leveraging the data of the large clinical trial conducted in Kolkata. We will first explore the magnitude of risk around cholera cases at different spatio-temporal scales. Based on this exploration, we will identify the suitable scale for the ring vaccination in that setting, and will estimate overall and indirect protective effectiveness (PE) of the oral cholera vaccine using a ring vaccination strategy.
Study type	Re-analysis of a cluster randomized clinical trial
Design	Cohort study
Subjects	All enumerated consenting subjects residing in Wards 29, 30 and 33 Eastern Kolkata, West Bengal, India.

Population size	Approximately 110,000 subjects in the study area
Vaccination period	First round - Dose 1 – 27 July to 13 August, 2006<br>Second round - Dose 2 – 27 August to 10 September, 2006
Agents	Bivalent, killed, whole-cell oral cholera vaccine and placebo
Primary end-point	Culture-proven *V. cholerae* O1 diarrhea episodes severe enough to require treatment in a health care facility
Study period	5 years post-receipt of second dose of either vaccine or placebo

3. Study Population

Two censuses were conducted in the original study area that includes all of ward 30 and part of ward 29 of the Kolkata Municipal Corporation. The cholera incidence in the study area was determined based on preliminary surveillance in the original study area. Subsequently, the geographic area was expanded in 2005 in preparation for the cholera vaccine trial. Table 1 below indicates the censuses performed in the study area. Each census captured the *de jure* population (defined as persons who stated their residence in the study area was their regular residence). The population database was updated following each census and the vital demographic events of the population were recorded.

Table 1. Censuses performed in different study wards

Ward	Census 1 Jan-Mar 2003	Census 2 Feb-Mar 2004	Census 3 Aug-Oct 2005	Census 4 Jan-Feb 2007	Census 5 Jun-Jul 2008	Census 6 Aug- Sep 2009	Census 7 Dec 2010-Jan 2011	Census 8 Oct- Nov 2011
Ward 29 (old)	√	√	√	√	√	√	√	√
Ward 29 (new)			√	√	√	√	√	√
Ward 30	√	√	√	√	√	√	√	√
Ward 33			√	√	√	√	√	√

4. Demographic Surveillance System (DSS)

Starting October 2007, a demographic surveillance system (DSS) was initiated to better define the corresponding dynamics in rates of birth, deaths, and migration in the population over time. Demographic events were recorded on a monthly basis during community health worker (CHW) visits.

5. Demographic Events

Out-migrations: Individuals who were present at the time of dose 1 but migrated out of the study area any time after dose 1 and during the period of follow-up under analysis.

Internal migrations: Individuals who were present at time of dose 1 but migrated from one place to another place within the study area after dose 1 and during the period of follow-up under analysis.

Deaths: Individuals who were present at time of dose 1 but died after dose 1 and during the period of follow-up under analysis.

Births: The births took place during the period of follow-up under the analysis.

6. Disease Surveillance

Nine clinics were established in the community to conduct the diarrheal disease surveillance. Private medical practitioners were encouraged to refer patients with diarrhea to these clinics. Additionally, surveillance was established in the two hospitals serving the study population. Patients from the study area were identified by use of household identification cards and a computerized database. Study physicians recorded pertinent clinical details on a structured clinical data form. Rectal swabs were obtained from all participants presenting with history of loose stools and transported in Cary-Blair media to a laboratory at NICED within 8 h of specimen collection. At the laboratory, rectal swabs were analyzed for *Vibrio cholerae* by serogroup, biotype, and serotype, by use of conventional methods. All patients whose swabs yielded *V cholerae* O1 or O139 were visited by a study team at their residence 7 days after the positive culture to verify that the individual whose identity had been given at the treatment center had visited on the recorded date of presentation.

7. Vaccination Period

First round (Dose 1): 27 July to 13 August, 2006

Second round (Dose 2): 27 August to 10 September, 2006

8. Follow-up Period

The last day of vaccination was 10 September 2006. This study will include cases reported in the project clinic/hospital from 1 October 2006 to until 5 years of post-dosing.

9. Definition of Principal Outcome Events

Diarrheal visit: A diarrheal visit is defined as an inpatient or outpatient visit for care of diarrhea in which the patient described:

- 3 or more loose or liquid stools; **or**
- At least 1 loose bloody stool; **or**
- 1-2 or an indeterminate number of loose or liquid stools and exhibited at least some dehydration

Diarrhea episode: A diarrheal episode is defined as follows:

- All diarrheal visit(s) for which the date of onset of symptoms for a diarrheal visit was less than or equal to 7 days from the date of discharge for the previous visit, constitute a single “diarrheal episode”.
- The onset of a diarrheal episode was defined as the day on which it was reported to have begun for the first visit of the episode.

Cholera episode: A cholera episode is defined as:

- A diarrheal episode in which no component visit was described as bloody diarrhea; **and**
- A fecal specimen from at least one component visit yielded *V. cholerae* O1 in the NICED laboratory; **and**
- An identity check performed 7 days after discharge for the visit in which *V. cholerae* O1 was isolated, confirmed that the person whose name was given at the treatment center had indeed sought care for diarrhea on the date of presentation

10. Other Definitions

Dynamic Population: The project dynamically updated the study area population using vital demographic events such as births, deaths, and migration-outs collected through routine demographic surveillance system, hence called dynamic population. This dynamic population (individuals residing in the study area at time of dose 1 or entered into the study any time after dose 1) will be included in the analysis.

Cluster: The original protocol was a cluster randomized trial. The unit of randomization was the premise, which is a household or group of households that usually share the same latrine(s) and water supply. The premise address is pre-assigned by the Kolkata Municipal Corporation. It may be one building with several flats or may be a group of huts.

Age eligible population: Persons from the study population aged 12.0 months and older at time of dose 1.

Randomized population: The randomized population includes individuals who were age-eligible and randomized to receive either the cholera vaccine or placebo.

Non-participants: Individuals who were present at time of dose 1 but not dosed. During routine demographic updates and census, individuals were identified who entered the population prior to dosing and therefore would have been eligible at the time of vaccination. These individuals are considered absent for registration at time of dose 1, and therefore considered part of the non-participants population. Individuals who were entered into the study area after vaccination, eventually did not receive the vaccine, will also be treated as non-participants in this analysis.

Population of 1-dose recipients: The population of 1-dose recipients includes individuals who drank and swallowed at least 1 dose of cholera vaccine or placebo.

Population of 2-dose recipients: The population of 2-dose recipients includes individuals who drank and swallowed 2 complete and correct doses (the full course) of either the cholera vaccine or placebo.

Absent for 2nd dose: Individuals who received only the 1st dose of the vaccine or placebo but did not return to take the 2nd dose of either vaccine or placebo despite being medically eligible for the second dose.

Incomplete dose: Individuals who did not ingest full amount of the administered dose. These individuals will be considered as non-participant in the analysis.

Wrong dose given: Individuals who were given the agent that is not assigned to him/her. These individuals will be considered as non-participants in the analysis.

Pre-dosing period: The pre-dosing period is the period on or before receipt of the first dose. Events whose onsets occur in this period will not be considered in the analysis.

Post dose 1 Interval: Post dose 1 interval begins on the date of the 1st dose and ends on the date of the 2nd dose. The interval range is 14 – 44 days. Events whose onsets occur in this period will not be considered in the analysis.

Post dose 2 Interval: Post dose 2 interval begins on the date of the 2nd dose and ends 30 September 2006. Events whose onsets occur in this period will not be considered in the analysis.

Post-follow up period: The time after the completion of the follow up period is defined as post-follow up period. Events whose onsets occur in this period will not be considered for analysis.

Vaccinees: Individuals who received two doses the oral cholera vaccine.

Non-vaccinees: Individuals who received at least one dose of placebo or the non-participants in the vaccination program.

Index case: A cholera case occurred anytime during the follow-up period in the study area

Index control: An index control will be randomly selected for each index case from the same study population matching by age-group (<5 years, 5-<15 years, and 15 years and above) at date of admission of its index case. The index controls are those who did not have cholera from 7 days prior to the onset date of its index case until the end of the surveillance period.

Contacts/neighbors: Individuals living in the study area within a specified distance range of the index case or index control.

Temporal/time scales: Temporal scales will be defined as first week (0-7 days), second week (8-14 days), and third week (15-21 days), etc. from the date of onset of index cases. We will go up to the 6th week from the date of onset of index cases.

Spatial/space scales: Spatial scales will be defined by 1st order of neighbor (individuals living 0-10.00 meters of the index case/control), 2nd order of neighbor (individuals living 10.01-15.00 meters of the index case/control), 3rd order of neighbor (individuals living 15.01-20.00 meters of the index case/control) and so on. Considering the size of study area, we will limit the orders of neighbor up to 50 meters.

Ring: We call the spatial as scale as ring, because we will include all people within the specified distance centering the index case/control.

Vaccine coverage: We will computed level of vaccine coverage within the ring, taking into account the two-dose recipients and all individuals presented in the ring at the onset of the illness of its index case. High and low vaccine coverage rings will be defined *post hoc*; rings with vaccine coverage (two doses) at 20th percentile of the population will be defined as low vaccine coverage ring and rings with vaccine coverage at 80th percentile of the population were defined as high vaccine coverage ring.

11. Data Analysis

We generated the following cohorts for the analysis in order to measure the risk for cholera around cholera cases as well as to measure vaccine effectiveness:

Cohort 1: All contacts of index cases excluding index cases

Cohort 2: All contacts of index controls excluding index controls

Cohort 3: All contacts of index cases living in high vaccine coverage rings excluding index cases

Cohort 4: All contacts of index cases living in low vaccine coverage rings excluding index cases

Cohort 5: Non-vaccinated contacts of index cases living in high vaccine coverage rings excluding index cases

Cohort 6: Non-vaccinated contacts of index cases living in low vaccine coverage rings excluding index cases

11.1. Evaluation of magnitude of risk around a cholera case

The first step of this analysis is to evaluate magnitude of risk around a cholera case. This will be achieved by evaluating the risk of cholera among contacts of index cases (cohort 1) relative to the contacts of index controls (cohort 2) at different space and time scale omitting index cases and index controls in the analysis. The cumulative incidence rate will be calculated in each cohort was follows:

Numerator: Cumulative total number of cholera cases in a given cohort.

Denominator: Cumulative total number of individuals in a given cohort.

In a second step we will compare these two risks using risk ratios. We will calculate the risk ratio of being a case occurring within a specified timeframe, t1 to t2, and within a specified distance range, d1 to d2, among contacts of index cases versus among contacts of index controls.

Statistical model: Both crude and multivariable logistic regression models will be used to calculate relative risk (RR) and 95% confidence intervals for the relative risk between contacts of index cases versus contacts of index controls.

11.2. Assessment of vaccine protective effectiveness (PE)

We will assess overall and indirect vaccine protective effectiveness

Overall Vaccine Protective Effectiveness (PE): The overall vaccine PE will be calculated by comparing the cumulative incidence rate among all contacts (irrespective of their vaccination status) of the index cases living in the high vaccine coverage ring versus the attack rate (cohort 3) among all contacts of the index cases living in the low vaccine coverage ring (cohort 4). The cumulative incidence rate in cohorts 3 and 4 will be calculated as described above for cohorts 1 and 2.

Statistical model for overall vaccine PE: Both crude and multivariable logistic regression models will be used to calculate relative risk (RR) and 95% confidence intervals for the relative risk between contacts

living in high vaccine coverage rings versus contacts living in low vaccine coverage rings. The adjusted relative risk will be transformed into vaccine PE as (1- RR) x 100%.

Indirect Vaccine Protective Effectiveness (PE)

The indirect PE will be calculated by evaluating the cumulative incidence rate among non-vaccinee (placebo or no dose recipient) contacts of the index cases living in high vaccine coverage rings (cohort 5) versus the attack rate among non-vaccinee contacts of the index cases living in low vaccine coverage rings (cohort 6). The cumulative incidence rate in cohorts 5 and 6 will be calculated as described above for cohorts 1 and 2.

Statistical model for indirect vaccine PE: Both crude and multivariable logistic regression models will be used to calculate relative risk (RR) and 95% confidence intervals for the relative risk between contacts living in high vaccine coverage rings versus contacts living in low vaccine coverage rings. The adjusted relative risk will be transformed into vaccine PE as (1- RR) x 100%.

11.3. This bias-indicator study

Since the high and low vaccine coverage rings will be defined *post hoc*, we will also evaluate PE against non-cholera diarrhea, defined as watery diarrhea (no blood in stools) and a faecal culture negative for *V cholerae* 01 in a bias indicator study. This bias-indicator study is designed to assess whether the results with respect to PE against cholera could be attributed to bias. An absence of PE in the bias-indicator study will be interpreted as suggesting an absence of bias in the PE against cholera. In bias indicator study, we will also calculate both overall and indirect vaccine protective efficacy.

Overall Vaccine Protective Effectiveness (PE): The overall vaccine PE against non-cholera diarrhea will be calculated by comparing the cumulative incidence rate of non-cholera diarrhea among all contacts (irrespective of their vaccination status) of the index cases living in the high vaccine coverage (cohort 3) ring versus the cumulative incidence rate of non-cholera diarrhea among all contacts of the index cases living in the low vaccine coverage ring (cohort 4). The cumulative incidence rate in cohorts 3 and 4 for

the bias-indicator study will be calculated as described above for cohorts 1 and 2, but with the outcome being non-cholera diarrhea.

Statistical model for overall vaccine PE: Both crude and multivariable logistic regression models will be used to calculate relative risk (RR) and 95% confidence intervals for the relative risk between contacts living in high vaccine coverage rings versus contacts living in low vaccine coverage rings. The relative risk will be transformed into vaccine PE as (1- RR) x 100%.

Indirect Vaccine Protective Effectiveness (PE)

The indirect PE will be calculated by evaluating the attack rate among non-vaccinee (placebo or no dose recipient) contacts of the index cases living in high vaccine coverage rings (cohort 5) versus the attack rate among non-vaccinee contacts of the index cases living in low vaccine coverage rings (cohort 6). The cumulative incidence rate in cohorts 5 and 6 for the bias-indicator study will be calculated as described above for cohorts 1 and 2, but with the outcome being non-cholera diarrhea.

Statistical model for indirect vaccine PE: Both crude and multivariable logistic regression models will be used to calculate relative risk (RR) and 95% confidence intervals for the relative risk between contacts living in high vaccine coverage rings versus contacts living in low vaccine coverage rings. The adjusted relative risk will be transformed into vaccine PE as (1- RR) x 100%.

12. Candidate variables for inclusion as covariates in the model

The factors known to be risk for cholera in the earlier study will be considered for the regression model. These are:

**Variable**	**Categories**
Age	In years (continuous)
Sex	0=female, 1=male
Vaccination status	0=non-participants/placebo recipient, 1= vaccine recipient
Individuals living in a household owning at least one luxury item	0=No, 1=Yes; items: Refrigerator, motorbike, television or washing machine

Distance (m) from the household to the nearest water body	Distance in meters (continuous)

13. Subgroup Analyses

We will conduct subgroup analyses of vaccine protection, which will be done on a strictly exploratory basis. The variables and their categories are given below.

**Variable**	**Categories**
Age at the date of admission of its index case	1=1-4.9 years, 2=5 years and above
Year of follow-up	1=Onset between 1 October 2006 and ZT+365<br>2=Onset between ZT+ 366 and ZT+730<br>3=Onset between ZT+731 and ZT+1095<br>4=Onset between ZT+1096 and ZT+1460<br>5=Onset between ZT+1461 and ZT+1825

ZT=Last date of 2nd round of vaccination

14. CONSORT for assembling the study population

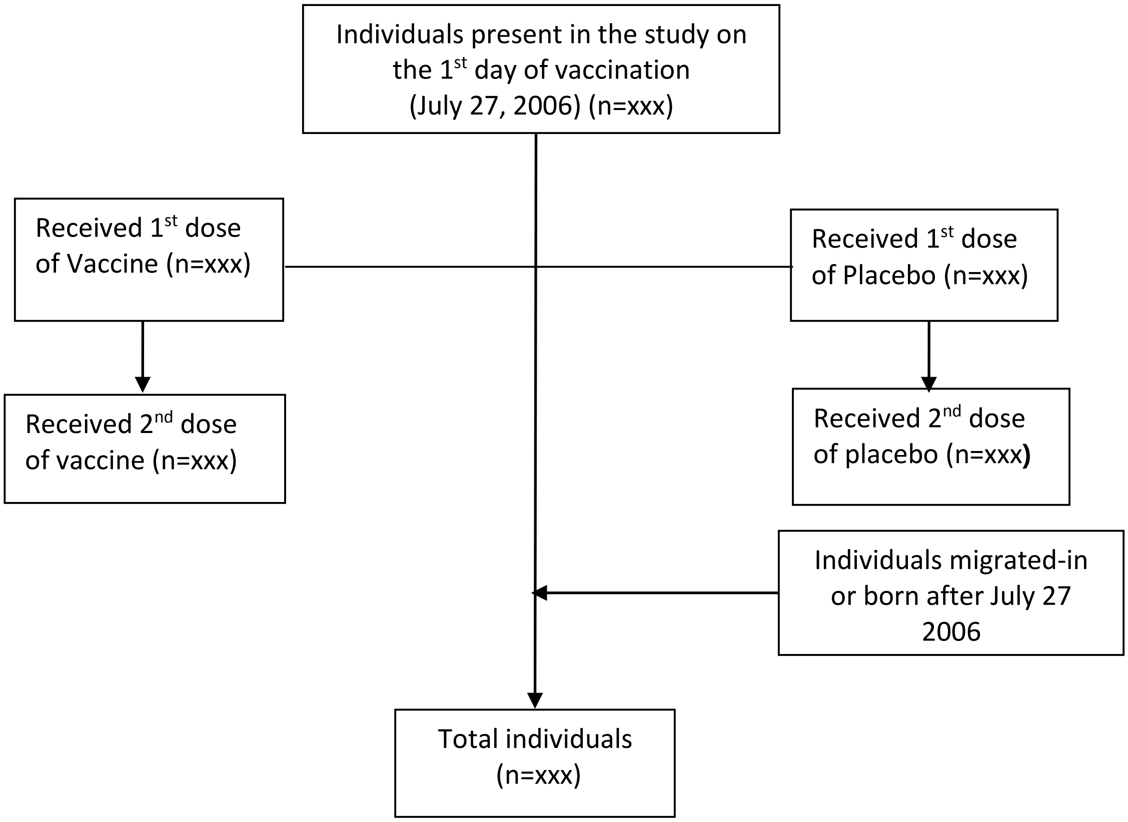

Supplement: S2 Text — (PDF) [file pmed.1002120.s009.pdf]
